# Innovation and progress towards TB elimination in New South Wales, Australia

**DOI:** 10.5588/ijtldopen.26.0047

**Published:** 2026-06-15

**Authors:** E.J. Donnan, J. Pett, C. Furlong, E. Ulbricht, P.D. Massey, V. Sintchenko, B.J. Marais

**Affiliations:** 1 Health Protection New South Wales, Health System Support Group, St Leonards, NSW, Australia;; 2 Sydney Infectious Diseases Institute (Sydney ID), The University of Sydney, Camperdown, NSW, Australia;; 3 Respiratory and Sleep Medicine Department, Liverpool Hospital, South Western Sydney Local Health District, Liverpool, NSW, Australia;; 4 Hunter New England Population Health, Hunter New England Local Health District, Newcastle, NSW, Australia;; 5 College of Medicine and Dentistry, James Cook University, Douglas, QLD, Australia;; 6 NSW Mycobacterium Reference Laboratory, New South Wales Health Pathology-Institute of Clinical Pathology and Medical Research, Westmead, NSW, Australia;; 7 Department of Infectious Diseases and Microbiology, The Children’s Hospital at Westmead, Westmead, NSW, Australia.

**Keywords:** tuberculosis, TBI, TB prevention, TB control, whole genome sequencing, contact investigation

## Abstract

**SETTING:**

Despite maintaining a low TB incidence (<10/100,000 population) for decades, progress towards TB elimination targets in Australia remains elusive.

**OBJECTIVE:**

We describe innovative approaches to improve TB care and control and programmatic components implemented to advance local TB elimination.

**RESULTS:**

Around 90% of people diagnosed with TB in New South Wales (NSW) were born outside Australia, with the greatest number of cases born in India, the Philippines, Nepal, and Vietnam. Routine whole genome sequencing allows local transmission tracking, and optimised data pathways provide better oversight of key performance indicators (KPIs), including the treatment of TB infection. Further efforts are underway to increase primary care involvement in TB preventive treatment (TPT) provision and to achieve stronger cultural engagement of vulnerable groups.

**CONCLUSION:**

Progress towards TB elimination in low-incidence settings requires renewed commitment and innovation, with careful consideration of local context. In NSW, key areas of innovation include improved transmission surveillance, enhanced data for KPI monitoring and optimised care of drug-resistant TB; however, reducing TB vulnerability, universal contact screening, and increased TPT coverage remain ongoing challenges.

Australia is a low TB incidence country, with a WHO incidence estimate of 6.2/100,000 population in 2023.^1^ The state of New South Wales (NSW) is the most populous, with 31.2% of the Australian population and the highest number of TB notifications (521 notifications; 32.1% of national notifications) in 2024.^2^ Overseas-born people comprise 31.0% of the population of NSW, with many born in high-TB-incidence countries. More than 90% (475/521; 91.2%) of people diagnosed with TB in NSW during 2024 were born overseas, with India (14.4%), the Philippines (14.0%), Nepal (11.5%), Vietnam (9.6%), and China (8.3%) the most common countries of birth.^3^ The 2015 WHO End TB Strategy had the ambitious goal to end the global TB epidemic by 2035.^4^ The three pillars of the strategy were: 1) integrated, patient-centred care and prevention, 2) bold policies and supportive systems, and 3) intensified research and innovation. The WHO action framework for TB elimination in low-incidence countries focused strongly on expanded TB disease and infection screening, combined with effective curative and preventive treatment.^5^ Australia aligned itself with the framework through the ‘Strategic Plan for Control of Tuberculosis in Australia, 2016–2020: Towards Disease Elimination’.^6^ This was then extended in the 2021–2025 strategic plan which incorporated an explicit focus on ‘zero local TB transmission’,^7^ an aspirational TB control target, and given the availability of new genomic tools to track TB transmission, the ability to monitor and prevent local epidemic spread.^8^

We previously reflected on key challenges that hinder TB elimination efforts in low-incidence settings such as NSW,^9^ and highlighted the limited awareness of TB among the public, and a general perception of low risk among Australian-born people and locally trained health care professionals. Migrants and hard-to-reach populations are overrepresented in TB notifications, whereas progress among First Nations peoples are complicated by embedded disadvantage and historical mistrust. Competing health priorities limit dedicated funding for elimination initiatives, which requires commitment and innovation, with careful consideration of local context.

Here we focus on innovative approaches to improve TB care and control and describe programmatic components implemented to advance towards TB elimination. In NSW, these approaches include innovation in ‘data for action’ and programmatic integration of routinely performed genomics, together with enhanced migrant screening, patient care, and contact tracing, as well as efforts to reduce TB vulnerability and maintain workforce capacity. It is important to recognise the decentralised nature and funding structure of NSW Health, where local health districts (LHDs) hold and disperse funding for medical services, including TB management and public health.

The NSW Population and Health Services Research Ethics Committee provided an exemption from ethical review for this manuscript (EFF25/23737).

## DATA FOR ACTION

Due to the significance of TB control as a public health function, NSW collects a large amount of epidemiological, clinical, and laboratory data (including genome sequencing of *Mycobacterium tuberculosis* isolates) for TB. Enhanced data capability, monitoring, and reporting to support policy development can maximise the value of collecting these data.

### Enhanced data capability

Revised national TB enhanced data specifications were implemented in 2024, which improved key data elements such as differentiating between relapse and reinfection, collecting phenotypic and genomic drug susceptibility test (DST) results, and better classification of well-recognised TB risk factors.^10^ Integration of *M. tuberculosis* genomic data has supported programme evaluation by differentiating true relapse cases that signify suboptimal TB care and identifying local TB transmission clusters indicating missed opportunities for prevention. Access to the Australian Immunisation Register data has facilitated better reporting on the use of the bacille Calmette-Guerin (BCG) vaccine administration. Work is underway to have a better record contact screening results and TB preventive treatment (TPT) outcomes across NSW.

NSW Health is currently in the process of implementing a state-wide digital patient record, with integration of electronic medical records, laboratory information management systems, and patient administration systems across state hospitals.^11^ This will benefit patients and clinicians, improve health system efficiency, and provide a unique opportunity to improve monitoring and evaluation via integration and/or data linkage.

### Defining key performance indicators

In preparation of this data revolution, the NSW TB Program developed preliminary state-wide key performance indicators (KPIs) (Table 1). Draft targets have been established based on either 1) programmatic goals (e.g., HIV testing conducted on >90% TB patients) or 2) incremental improvement. These KPIs and targets are open to ongoing refinement but serve an important monitoring function by the NSW Tuberculosis Advisory Committee (TBAC). TBAC consists of experienced clinical and public health physicians, nurses and a pharmacist, as well as Health Protection NSW TB Program personnel. TBAC performs quarterly reviews of epidemiological and KPI data. This is complemented by formal annual KPIs review including TB-related mortality and serious adverse events, with careful consideration of potential preventable factors. Contact screening completeness and outcome data are provided to LHD TB clinics, and public health and clinical governance units for performance review.

**Table 1.** Overview of provisional key performance indicators (KPIs)^A^ for enhanced TB control in NSW, Australia.

Thematic area	Key performance indicator
TB disease rates	TB notification rate (per 100,000 per year)
	TB notification rate in Aboriginal and Torres Strait Islander people
	TB notification rate in Australian-born non-Indigenous Australians
Reporting	Proportion of cases notified within 4 months of first health contact
TB service and prevention	Proportion of cases (all and PTB-specific) commenced on TB treatment within 7 days of notification
	Median days from notification to effective treatment for MDR-TB cases
	Proportion of cases with HIV testing results available
	Number of children <5 years with TB disease eligible for BCG vaccination, but without a history of BCG vaccination
	Proportion of laboratory confirmed pulmonary cases with ≥1 contact identified
	Proportion of contacts identified of microbiologically confirmed PTB cases that completed screening
	Proportion of household contacts <5 years of microbiologically confirmed PTB cases that completed screening
	Proportion of household contacts <5 years of age commenced on TPT
Laboratory services	Proportion of culture confirmed cases with DST
	Proportion of culture confirmed cases sequenced
Local TB transmission	Proportion of sequenced cases that are part of an NSW cluster (≤5 SNP cut-off) in a single year
	Proportion of sequenced cases that are part of an NSW cluster of concern^B^
Outcomes	Successful outcomes^C^ for MDR-TB and non-MDR-TB cases
	Avoidable TB-related deaths^D^

A national ‘cluster of concern’ definition is being finalised with the availability of routine genomics in all states and territories.

BCG = bacille Calmette-Guerin; PTB = pulmonary TB; MDR-TB = multidrug-resistant TB (resistance to isoniazid and rifampicin); NSW = TPT = New South Wales; TB preventive therapy; DST = drug susceptibility testing; SNP = single nucleotide polymorphism.

A KPIs have not been formally endorsed and currently under assessment.

B Cluster (identified using ≤5 SNP cut-off) with ≥5 members over a 5 year ‘rolling period’, suggesting ongoing transmission within NSW.^12^

C Include the outcomes ‘cured’ and ‘completed treatment’. Outcomes not assessed include transferred overseas and died of cause other than TB.

D As assessed by clinician expert panel.

The NSW TB Program monitors large-scale (≥25 contacts) contact screening events, particularly those involving health care facilities or educational institutions, those that may cause public concern, or cross state and/or international jurisdictional borders.^13^ Over the past 10 years (2015–2024), there were around 24 (range 7–41) large screening events per year, placing a significant burden on health care resources and demonstrating the need for surge capacity staffing that can also be drawn on to contain other infectious disease outbreaks.

### Improved TB reporting

TB reporting procedures have been streamlined. Given the decentralised nature and funding structure of NSW Health where LHDs fund local TB services, standardised reports are made available to local TB services and public health units. These reports include epidemiological and activity overviews, as well as KPI assessments with most components updated monthly, otherwise quarterly for migration and vaccination data. Reported activity includes the number of people diagnosed with TB, contact screening cascade of care results, number of people treated for TB infection (TBI), migrant referrals, BCG requests and vaccine administrations, as well as LHD-specific KPIs and sentinel events. An annual surveillance report provides a more comprehensive state-wide overview, which is now publicly available as a user-friendly interactive website report.^14^

## GENOMICS INNOVATION

In 2016, routine whole genome sequencing (WGS) of all *M*. *tuberculosis* isolates was introduced as part of an NSW Health Transitional Research Grant and subsequently funded by NSW Ministry of Health as a routine service (2018 onwards). The NSW Mycobacterium and Pathogen Genomics Reference Laboratories, hosted by NSW Health Pathology, undertake prospective sequencing with results routinely available within 1–2 weeks of an isolate becoming culture positive. Cultures recovered from both pulmonary and extra-pulmonary patients are included in this genomic surveillance. The availability of WGS-based drug susceptibility testing (DST) prediction offers a significant time saving compared to traditional phenotypic DST and provides a comprehensive overview of mutations conferring potential resistance to clinically relevant TB drugs.^15^ WGS has both clinical and public health utility, including comprehensive drug-resistance surveillance, identification of laboratory cross-contamination, and differentiating relapse from reinfection.^16^

### Successful integration of genomics into TB care and control

Clinician education and regular structured communication between the sequencing laboratory, the NSW TB Program, and frontline TB nurses together with regular joint review of genome sequencing, epidemiology, and clinical case management findings have been crucial to unlock the full clinical and public health utility of routine WGS. Work is underway to ensure all genomic DST and clustering results are readily captured in both the laboratory and clinical information systems. Since 2018, there have been iterative improvements to the integration of genomic, clinical, and public health data, with genomics data now included in quarterly internal TB control reports, KPI data, and public-facing annual surveillance reports. The identification of likely local TB transmission events in NSW has provided an opportunity for improving contact tracing and better targeted disease control efforts to minimise and rapidly terminate local transmission chains.^15^

There is scope for further improvement in the utilisation of WGS data for clinical and public health purposes. NSW Health Pathology is currently building routine reporting from WGS into the state Laboratory Information System, which will be available for all clinicians involved in the care for TB patients across the state. Programmatic monitoring of disease relapse, local transmission, and missed opportunities for prevention will help to identify modifiable risk factors that could improve clinical and public health processes. The TB programme has been also supporting sequencing data sharing for investigations of cross-jurisdictional clusters and cases associated with clusters examined overseas.

## MIGRANT SCREENING

All visitors and migrants to Australia must meet strict public health requirements to protect the Australian community from infectious disease, with specific consideration of TB risk, with a specific TB disease declaration required on the visitor arrival card.^17^

### Migrant referrals

Immigration medical examinations (IMEs) are performed on all migrants from high-TB-incidence (≥40/100,000 population) countries who plan to stay in Australia for a period of 6 months or more. An IME consists of a detailed exposure history and clinical examination, as well as a chest radiograph (CXR) and sputum culture or Xpert MTB^®^ (if aged >10 years). In addition, an interferon-gamma release assay (IGRA) or tuberculin skin test (TST) is performed for migrants intending to work, study, or train in health care, aged care, or disability. Children aged 2–10 years have an IGRA or TST performed, and a CXR if they test positive. Migration screening may either be undertaken pre-migration (offshore), or in Australia if people are applying for a new or extended visa (onshore). In 2024, almost 20% of people diagnosed with TB in NSW (102, 19.6%) had been referred through migration screening. Of these, 32 people (31.4%) were referred following concern during offshore screening, but without detectable active disease at the time. The majority (70, 68.6%) were already in Australia and identified from an IME for an application for a new or extended visa. Migrant screening policies in Australia and its associated challenges have been previously discussed.^9^

### Referral follow-up

Recent improvements in NSW Health processing of migrant TB referrals allow better tracking to assess referral trends and outcomes. Apart from TB testing and monitoring, migrant screening referrals also allow clinics to provide TB education and to encourage TPT uptake, where relevant. The feasibility of primary care general practitioners (GPs) becoming involved in migration TB screening is being examined to improve access and ensure that specialist TB services are not overwhelmed. Extensive work has also been undertaken in collaboration with the Department of Home Affairs to develop objective and efficient data-driven screening approaches.^18^ Enhanced data collection and anonymous data linkage is important to improve data-driven decision making.

## PATIENT CARE

Significant efforts to improve and individualise patient care have included providing expert panel review for patients with complex presentations or drug-resistant TB, patient-centred treatment adherence monitoring, and better access to child-friendly and other key medications.

### Expert panel review of complex cases

All multidrug- and rifampicin-resistant TB (MDR/RR-TB) cases are routinely presented to an expert panel presented by the treating physician and district clinical nurse consultant.^19^ Other complex cases are reviewed by request. The panel, consisting of respiratory and infectious disease physicians, and public health and reference laboratory representatives, considers optimal clinical and public health management in line with current evidence-based practice and professional experience. In 2024, there were panel discussions for eight MDR/RR-TB patients and nine complex cases with issues such as other polyresistance, significant tolerance issues/side effects and management of paradoxical reactions.

### Patient-centred treatment

Previously, the policy in NSW was treatment administration for all people with TB disease should be by directly observed therapy.^20^ The policy has now been updated to ensure an individualised, patient-centred approach to support optimal treatment adherence is required.^19^ TB clinics have mechanisms to monitor treatment adherence in a manner that continues to minimise community risk from TB transmission and ensure successful treatment completion, but is also limits financial hardship and is minimally restrictive to patients.

### Access to TB medications

Due to stringent regulatory requirements and the cost associated with registering medicines with Australia’s Therapeutic Goods Administration (TGA), some TB drugs are not formally registered or available via standard pathways. Rifapentine, most second-line drugs, and all fixed-dose combination tablets (FDCs) are unregistered and require use of the TGA’s Special Access Scheme (Table 2).^21^ To facilitate access to some of the drugs, NSW Health has worked with the Stop TB Partnership Global Drug Facility via a not-for-profit corporate arrangement. This has resulted in access to the child-friendly water-dispersible FDCs, and more recently rifapentine–isoniazid FDCs for TPT.

**Table 2.** Anti-TB medications Australian Therapeutic Goods Administration (TGA) registration status and Special Access Scheme (SAS)^A^ pathways, Australia.

TGA status	Drug	SAS pathway
Registered	Isoniazid (100 mg only)	N/A
	Rifampicin	N/A
	Ethambutol (400 mg only)	N/A
	Pyrazinamide	N/A
	Linezolid	N/A
	Meropenem	N/A
Unregistered (authorised prescriber or SAS)	Bedaquiline	B
	Pretomanid	B
	Clofazimine	C
	Cycloserine	B
	Ethionamide/Prothionamide	B
	Delamanid	B
	Para-aminosalicylic acid	B
	Rifapentine	B
	Adult FDC (HREZ/HR)^B^	B (treatment) or C (HR for TBI only)
	Paediatric FDC (HRZ/HR)^B^	B (treatment) or C (HR for TBI only)
	Isoniazid–rifapentine FDC^B^	C

FDC = fixed-dose combination tablets; HREZ = soniazid, rifampicin, ethambutol, and pyrazinamide (also known as RIPE); HR = isoniazid and rifampicin; HRZ = isoanizid, rifampicin, pyrazinamide; TBI = TB infection; NSW = TPT = New South Wales.

A SAS Pathways^21,22^ are: A – notification pathway for patients seriously ill with a condition that is reasonably likely to lead to death within a year; B – approval pathway (2–5 days); C – notification pathway for select unapproved therapeutic good is on the established history of use list.

B Items NSW obtains via the Stop TB Partnership Global Drug Facility.

### Decentralised GP-led services

GPs currently contribute to health care worker screening in NSW, with follow-up attended to within the public hospital system. However, there is limited capacity within specialist respiratory or infectious disease settings for TPT management.^23^ TPT provision within primary care settings has been trialled in the USA, the UK, Canada, and in Victoria, Australia,^23–25^ with commitment to examine viable implementation models in NSW.^26^ Upskilling of GPs to undertake further aspects of TB management, including health care worker and migrant screening, and to provide TPT, would improve general TB awareness which may reduce instances of diagnostic delay, and potentially deliver a more person-centric service and support to the public hospital system.^27,28^

## ADDRESSING VULNERABILITY

All services related to the diagnosis and treatment of TB disease or infection are provided free of charge to patients within the NSW public health system, regardless of eligibility for Medicare (Australia’s universal health care system) or visa status.^19^ However, other costs such as time off paid work and transportation to health care facilities are not subsidised, which is problematic for people who only get paid for work done. Some governmental supports can be facilitated via hospital social work departments for Australian citizens or permanent residents. Limited non-governmental organisational support may be available for people on other visas or with no visa. No data are currently available on the indirect costs of TB in NSW.

### Culturally and linguistically diverse communities

**Figure 1.**
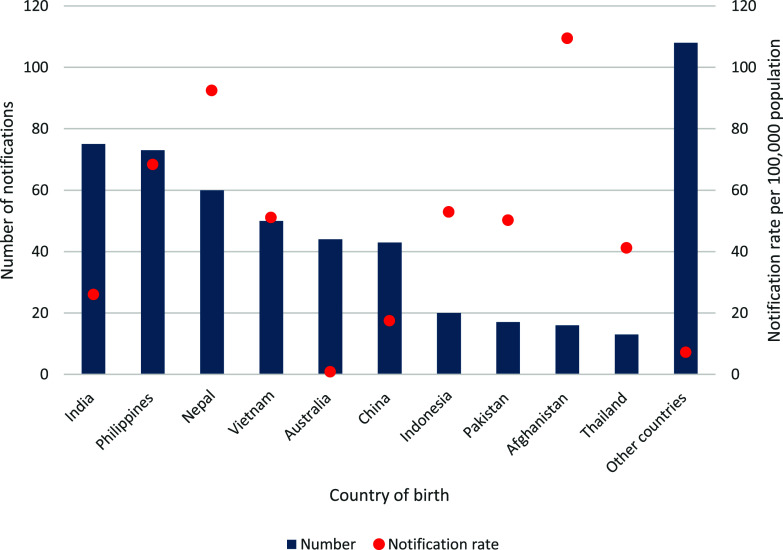
Number of TB notifications and notification rate by country of birth in New South Wales, Australia (2024).

With 91.2% of people diagnosed with TB in NSW in 2024 born overseas (Figure 1), and 58.6% recording a primary language other than English, engaging culturally and linguistically diverse communities in care is crucial. An understanding of cultural context is essential, and individual approaches may be required to provide patient-centred care with consideration given to language and social context. Multilingual TB staff are employed in TB clinics in most metropolitan TB clinics. National Accreditation Authority for Translators and Interpreters (NAATI)-certified interpreters are available for most languages. Written TB resources are available in 17 languages for TB, TBI testing and treatment, and BCG vaccination. Significant efforts have been made to transform long, complex fact sheets into plain English and incorporate pictures and icons.

Further work to better engage with diverse migrant communities, through better targeted and culturally appropriate engagement, would be beneficial. This could include reviewing available surveillance and clinical data, engaging with TB survivors and community leaders to explore how health-seeking behaviours and health system engagement can be improved, addressing knowledge gaps and stigma surrounding TB, and exploring co-creation of communication of health care messages around TB with specific communities.^29^

### Aboriginal and Torres Strait Islander people

Australia’s first nation people, Aboriginal and Torres Strait Islander people, are overrepresented in TB notification in Australian-born people in NSW.^30^ This overrepresentation is likely a consequence of embedded disadvantage, health care access challenges in some areas, and mistrust.^7^ In response to a protracted outbreak of TB in regional NSW that spanned more than 20 years,^31,32^ significant cultural engagement work was undertaken with Aboriginal people and organisations in those regional areas, recognising the importance of care that maintained Aboriginal people’s connection to country. Participatory action research methods were used partnering with local TB clinicians and environmental health teams to improve housing health hardware and nutrition alongside routine TB clinical care.^33^ TBI screening was undertaken with an Aboriginal Community Controlled Health Service.^34^ Further collaboration with Aboriginal and Torres Strait Islander–led health services and communities is necessary to respond to TB inequity, the social determinants of health, and the historical, cultural, social, and structural processes relevant to TB care and control.

## CONTACT INVESTIGATION

**Figure 2.**
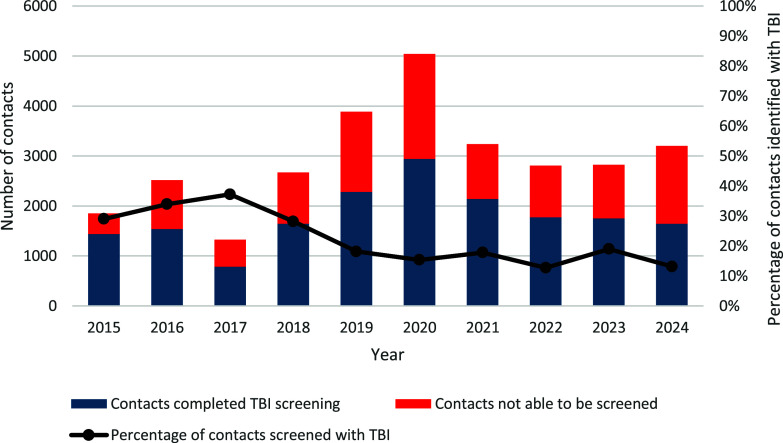
Number of TB contacts* identified and screened for TBI, and proportion identified with TBI, in New South Wales, Australia (2015–2024). *Close contacts of people diagnosed with microbiologically confirmed pulmonary TB. TBI = TB infection.

Timely screening of all close contacts is considered a key action for low-incidence countries towards TB elimination.^5,35^ Contact screening for TB is a public health requirement, and guidelines are in place to support this.^13,19^ The completeness of contact screening has remained relatively constant in the past 10 years (average 62.0% completeness range 51.3%–77.9%) (Figure 2).

Updated state-wide guidelines on contact investigation and screening were introduced in 2019, which led to increased identification of contacts, particularly contacts of people with smear negative, but culture or Xpert positive pulmonary TB. TPT uptake in contacts found to have TBI has increased from 32% in 2015 to a peak of 56% in 2022, with a slight decrease in 2024. Until relatively recently, many clinicians did not offer TPT to people with documented TBI from historic exposure (such as being born or having lived in a high-TB-incidence country). TPT provision to young and vulnerable children (<5 years) with documented TB exposure or infection was more consistent, and the full cascade of care in these children is tracked. Better guideline awareness, alongside local TB transmission mapping that identifies secondary TB cases amongst untreated contacts, is having a positive impact on TPT prescribing and uptake.

### Opportunities for improvement

Currently, only aggregated contact screening data are collected. A more detailed exploration of TB screening outcomes, including TPT uptake and completion, should identify further opportunities for improvement. It is anticipated that new insights will guide better engagement methods in specific local contexts and congregate settings where screening completeness may be lower, with further insights into uptake of TPT. TB contact management currently occurs in hospital outpatient and TB clinic settings. The contribution of primary care GPs to support TB contact management, either as a primary or follow-up mechanism, is being considered. Important barriers include GPs limited TB knowledge, funding of IGRAs (only available for Medicare-eligible people), Special Access Scheme requirements and difficulty accessing rifapentine, and rifamycins used for TPT are not covered by the Australian Pharmaceutical Benefits Scheme.

## TB WORKFORCE

Maintaining a highly skilled and dedicated public workforce for TB care and control activities is critical. The Australian National Tuberculosis Advisory Committee published the essential components of a jurisdictional TB Control Program in Australia in 2014,^36^ which emphasised the importance of maintaining a skilled and dedicated TB workforce. The new ‘Tuberculosis Workforce Policy and Development Framework’ describes the roles and key skills of the multidisciplinary TB workforce, the workforce structure of public TB programmes in Australia, as well as the priorities and strategies for workforce training and development.^37^

### Connectedness and competency

**Figure 3.**
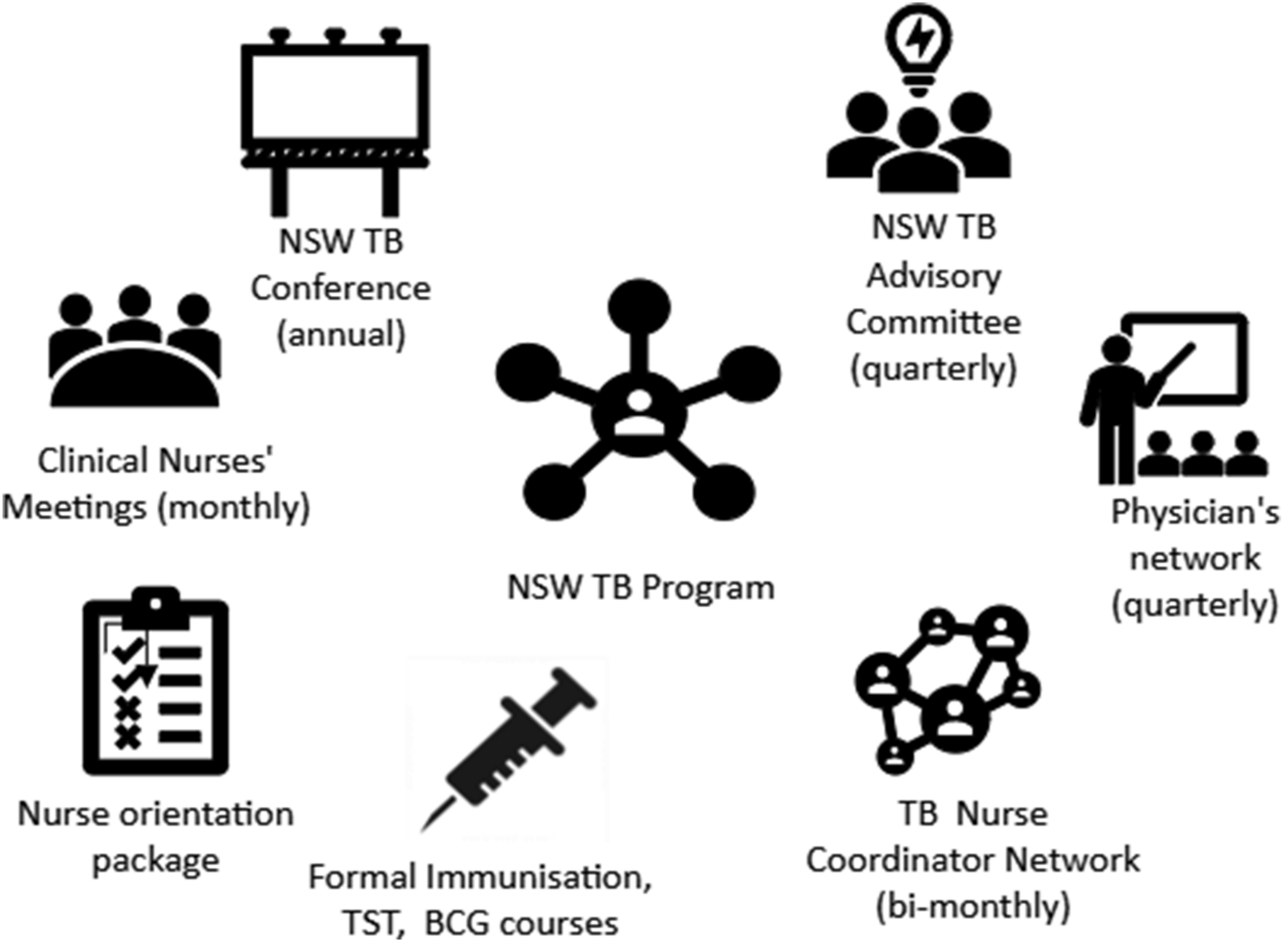
TB-specific education opportunities and networks within NSW Health. *The NSW TB Program operates within Health Protection NSW (part of the NSW Ministry of Health). NSW = New South Wales; TST = tuberculin skin test; BCG = bacille Calmette-Guerin.

The NSW TB Program has worked to maintain strong TB-specific networks and education opportunities (Figure 3). These include TB nursing clinical competency standards and skill sets, formal TST and BCG courses for nurses, and a state-wide new TB nurse orientation module. Monthly clinical TB nurses’ education meetings are important to exchange knowledge and learn from local experience and to maintain a sense of connectedness and camaraderie among geographically dispersed nurses. The NSW TBAC brings together respiratory, infectious diseases, and public health physicians, as well as nurses and a pharmacist with TB experience to set the strategic direction and provide expert advice. A TB Network SharePoint page and TB physician and TB nurse mailing lists are maintained to seek guidance and communicate information such as policy changes, treatment updates, and drug availability. An annual NSW TB conference including medical, nursing, laboratory, public health, and allied health workers, provides a valuable vehicle for knowledge exchange and community building. The Australasian Clinical Tuberculosis Network (ACTNet) providing clinical and research webinars to a national audience,^38^ and annual research seminars are coordinated by the Centre for Research Excellence in TB Control (www.tbcre.org.au) funded by the National Health and Medical Research Council.^39^

## CONCLUSION

Globally, progress towards TB elimination in low-TB-incidence countries has been limited. A robust data collection system is essential for enhanced surveillance, better service planning, and KPI monitoring. Advances in TB genomics offer exciting opportunities to personalise treatment and implement better targeted interventions to limit local TB transmission.^8^ The introduction of a single electronic medical record and full integration of TB genomics into public health data systems present exciting opportunities for optimal data integration. However, optimal TB care and control ultimately depend on a dedicated and passionate network of health professionals and effective community engagement.
